# Predictors of Prolonged Stay in the Intensive Care Unit following Cardiac Surgery

**DOI:** 10.5402/2012/691561

**Published:** 2012-06-27

**Authors:** Rokeia Eltheni, Konstantinos Giakoumidakis, Hero Brokalaki, Petros Galanis, Ioannis Nenekidis, George Fildissis

**Affiliations:** ^1^Cardiac Surgery Intensive Care Unit, “Evangelismos” General Hospital of Athens, 45-47 Ipsilantou Street, 10676 Athens, Greece; ^2^Faculty of Nursing, National and Kapodistrian University of Athens, 123 Papadiamantopoulou Street, 11527 Athens, Greece; ^3^Centre for Health Services Management and Evaluation, Faculty of Nursing, National and Kapodistrian University of Athens, 123 Papadiamantopoulou Street, 11527 Athens, Greece; ^4^First Cardiothoracic Surgery Department, “Evangelismos” General Hospital of Athens, 45-47 Ipsilantou Street, 10676 Athens, Greece

## Abstract

The prediction of intensive care unit length of stay (ICU-LOS) could contribute to more efficient ICU resources' allocation and better planning of care among cardiac surgery patients. The aim of this study was to identify the preoperative and intraoperative predictors for prolonged cardiac surgery ICU-LOS. An observational cohort study was conducted among 150 consecutive patients, who were admitted to the cardiac surgery ICU of a tertiary hospital of Athens, Greece from September 2010 to January 2011. Multivariate regression analysis revealed that patients with increased creatinine levels preoperatively (odds ratio (OR) 3.0, *P* = 0.049), history of atrial fibrillation (AF) (OR 6.3, *P* = 0.012) and high EuroSCORE values (OR 2.6, *P* = 0.017) had a significant greater probability to stay in the ICU for more than 2 days. In addition, intraoperative hyperglycemia (OR 3.0, *P* = 0.004) was strongly associated with longer ICU-LOS. In conclusion, the high perioperative risk, the history of AF and renal dysfunction, and the intraoperative hyperglycemia are significant predictors of prolonged ICU stay. The early identification of patients at risk could allow the efficient ICU resources' allocation and the reduction of healthcare costs. This would contribute to nursing care planning depending on the availability of healthcare personnel and ICU bed capacity.

## 1. Introduction

Intensive care requires not only the use of sophisticated equipment but also highly skilled and dedicated healthcare staff. As such, the intensive care unit (ICU) takes a significant proportion of the total healthcare cost, and therefore patients with prolonged ICU length of stay (LOS) can have serious cost implications. Moreover, patients with prolonged ICU-LOS can also lead to a shortage of ICU beds and result in operations being cancelled [1].

Regarding the population of cardiac surgery patients, the prediction of their ICU-LOS is a fact of great significance, based on the need for containing the burgeoning costs involved in cardiac operations, saving resources and ICU costs [2]. In addition, the cardiac surgery ICU-LOS is a significant healthcare index that has been correlated with poor patient outcome, such as increased expenditure and higher morbidity and mortality rates [3]. Furthermore, the ICU-LOS is a valuable indicator of the quality and the effectiveness of the provided care [4].

The above data could interpret the scientific interest for the early prediction of the length of hospitalization in cardiac surgery ICUs. Various predictive models for prolonged ICU stay after cardiac surgery have been developed, during the last two decades [5, 6], and several papers have attended preoperative risk factors associated with prolonged ICUstay [1]. These efforts are now more than ever in season and necessary because of the global financial crisis, that has affected negatively the healthcare systems' operation, worldwide, and demands a more efficient use of the ICU resources.

The aim of this study was to identify the preoperative and intraoperative predictors for prolonged ICU-LOS, among cardiac surgery patients. This identification could lead to the early recognition of the high-risk patients for longer ICU hospitalization and, also, could provide an important aid to nursing administrators and leaders for the appropriate resource planning and effective choice of the operation list, with the low-risk patients being scheduled for surgery before the high-risk candidates.

## 2. Methods

### 2.1. Design and Settings

We conducted an observational cohort study from September 2010 to January 2011 among 194 patients who were admitted to the 8-bed cardiac surgery ICU of a general, tertiary hospital of Athens, Greece. The inclusion criteria into the study were a priori defined as follows: (a) patient age ≥18 years old and (b) a minimum ICU-LOS of 24 hours. Based on the predefined inclusion criteria 44 patients were excluded from the study, and 150 met the inclusion criteria and constituted our study sample.

### 2.2. Data Collection

A short structured questionnaire on basic sociodemographic and clinical patient characteristics was used for data collection purposes. This tool was created by the researchers of this study. The collected sociodemographic data included patient age, gender, height, weight, and body mass index (BMI). In addition, the collected clinical characteristics were the history of chronic pulmonary disease and diabetes, the type of surgery, the preoperative serum creatinine (Cr) levels, the blood glucose levels during the intraoperative period, the use of intraaortic balloon pump (IABP) preoperatively, the application of cardiopulmonary bypass (CPB), the preoperative ejection fraction of the left ventricle (EFLV), the duration of surgery and CPB, the presence of atrial fibrillation (AF) preoperatively, the emergency procedure, the ischemic time, the transfusion of red blood cells (RBCs), the EuroSCORE values and the ICU-LOS. We defined the ICU-LOS as the duration of the hospitalization of patients from their admission to the ICU until their discharge.

EuroSCORE is the most valid and reliable stratification model for predicting the perioperative risk of cardiac surgery patients in North America, Europe, and Japan [7]. It was developed between 1995 and 1999 and was first introduced into clinical practice in 1999, while the logistic algorithm of EuroSCORE is available since 2003 [8]. It includes three wide categories of risk factors: the patient- (age, female sex, chronic pulmonary disease, extracardiac arteriopathy, neurological dysfunction disease, previous cardiac surgery, serum creatinine preoperatively, active endocarditis, critical preoperative state), the cardiac- (unstable angina, left ventricular dysfunction moderate: left ventricular ejection fraction (LVEF) 30–50% or poor: LVEF <30%, recent myocardial infarct (<90 days), pulmonary hypertension, and the operation- (emergency, other than isolated coronary artery bypass grafting, surgery on thoracic aorta, postinfarct septal rupture) associated factors. The sum of the various risk factors results in a total score which represents the predicted probability of mortality and ranges between 0 and 100% (logistic EuroSCORE) [8, 9].

One of the researchers, the same each time, obtained the data based on the review of both medical and nursing patient records. EuroSCORE was calculated for each patient based on data collected by the hospital records. In total, we carried out 150 EuroSCORE measurements. Finally, the measurement of the serum glucose levels was carried out for each patient, intraoperatively, immediately after the induction of anesthesia and was repeated every 30 minutes.

### 2.3. Statistical Analysis

Continuous variables are presented as median (interquartile range, IR), while categorical variables are presented as absolute and relative frequencies. The Kolmogorov-Smirnov test and graphs (histograms and normal Q-Q plots) were used to test the normality of the distribution of the continuous variables. Distributions of continuous variables were extremely skewed and were converted into binary variables using median values as the criterion for separation. For BMI the criterion for separation was the international limit of 24.9 kg/m^2^. For serum Cr levels the cut-off point was the 1.4 mg/dL, a value that constitutes the limit between the normal and the increased Cr levels of the biochemistry laboratory of the hospital in which our study was carried out. In addition, the cut-off for prolonged ICU-LOS (2 days) was chosen based on the median value and the literature data [3, 10].

Chi-square test and Fisher's exact test were used to identify differences between groups. Kendall's tau rank correlation coefficient (*r*) was used to estimate correlation between continuous variables. Variables that were statistically significant (*P* < 0.05) in bivariate analysis were entered into the backward stepwise multivariate logistic regression analysis with ICU-LOS as dependent variable. Multivariate analysis was used to control potential confounding variables. Only variables with 95% confidence interval for adjusted odds ratio that did not cross 1 were considered to have an independent and significant association with ICU-LOS. Criteria for entry and removal of variables were based on the likelihood ratio test, with entrance and removal limits set at *P* < 0.05 and *P* > 0.05. We estimated crude and adjusted odds ratios (ORs) with 95% confidence intervals (CIs) for the predictive factors related to the ICU-LOS. A correlation matrix was assessed prior to conducting the multivariate logistic regression analysis to check for collinearity among the independent variables. Kendall's tau rank correlation coefficient (*r*) was used to estimate correlations between variables. All tests of statistical significance were two-tailed, and *P*-values of less than 0.05 were considered significant. Statistical analysis was performed using the Statistical Package for Social Sciences software (IBM SPSS Statistics 19.0 for Windows, SPSS Inc., Chicago, IL, USA).

### 2.4. Ethical Considerations

Permission to conduct this study was obtained by the ethical committee of the hospital. Researchers received patients' consent after being informed about the type and purpose of the study. Each potential subject adequately informed of the aims, method, the anticipated benefits, and potential risk of the study. The potential subject informed of the right to refuse to participate in the study or to withdraw consent to participate any time without reprisal. The investigation was carried out in accordance with the ethical standards of the responsible institutional committee for human experimentation and with the Helsinki Declaration of 1975, as revised in 2008. Precautions took place to protect the privacy of research subjects and the confidentiality of their personal information. The methods of the study were restricted to observation and recording patient data and no part of the standard care was omitted.

## 3. Results

Demographic and perioperative characteristics of the sample are shown in Table 1. The majority of patients were males (68%), and the median age was 66 (IR 15) years old. Most patients had either received a coronary artery bypass grafting (44.7%) or had undergone a valve disease surgery repair or replacement (27.3%). 17.3% and 48% of patients had serum Cr levels higher than 1.4 mg/dL and blood glucose levels higher than 130 mg/dL, respectively. Preoperative AF was presented in 11.7% of patients, while the median logistic EuroSCORE value was 5.4 (7.3) %. The median ICU-LOS was 2 (IR 3) days, and the overall 30-day mortality was 13.3%.

Table 2 provides the main predictive factors' (including demographic characteristics and perioperative-related factors) distribution and their relation with ICU-LOS (below or above median). The preoperative serum Cr >1.4 mg/dL, the mean intraoperative blood glucose levels >130 mg/dL, the duration of surgery and the duration of CPB longer than 250 and 125 min, respectively, the presence of AF preoperatively, the emergency procedure, the transfusion of RBC and the logistic EuroSCORE values >5.4% were associated with ICU-LOS more than 2 days at the level of 5% (*P* < 0.05) in bivariate analysis.

Table 3 summarizes the main findings of the logistic regression analysis. A correlation matrix was assessed prior to conducting the multivariate logistic regression analysis to check for collinearity among the independent variables. Because duration of surgery and duration of cardiopulmonary bypass were strongly correlated (*r* = 0.66, *P* < 0.001), we chose to include only the duration of surgery in the multivariate model. We found a positive association between perioperative risk (high EuroSCORE) and ICU-LOS (OR = 4.3, 95% CI = 2.2–8.4, *P* < 0.001) while this association remained statistically significant after adjusting for gender, age, BMI, chronic pulmonary disease, diabetes, type of surgery, preoperative IABP, CBP, EFLV, duration of surgery, cardiopulmonary bypass time, emergency, ischemic period, and RBC (OR = 2.6, 95% CI = 1.2–5.6, *P* = 0.017). In addition to the above, patients with preoperative serum Cr >1.4 mg/dL had an almost 3 times greater probability to have ICU-LOS >2 days (OR = 3, 95% CI = 1–8.7, *P* = 0.049), and also patients with mean intraoperative blood glucose >130 mg/dL had an almost 3 times greater probability for longer ICU-LOS (OR = 3, 95% CI = 1.4–6.4, *P* = 0.004). Finally, patients with AF preoperatively were associated with an almost 6.3 times greater probability of having an ICU-LOS of more than 2 days.

## 4. Discussion

The main findings of our study were the strong association of three preoperative (serum Cr levels, preoperative AF, and logistic EuroSCORE) and one intraoperative factor (glucose levels) with the prolonged ICU-LOS of cardiac surgery patients. In particular, patients with preoperative renal dysfunction, high perioperative risk, history of AF or high mean glucose levels intraoperatively have greater possibility to stay in the cardiac surgery ICU more than 2 days.

As aforementioned, the increased serum Cr levels, preoperatively, were statistically significantly correlated with prolonged ICU hospitalization. In line with our findings, Herman et al. [11], in a recent study of 3489 CABG patients in a hospital of Canada, concluded that the history of renal failure is among the independent predictors for prolonged hospitalization into the intensive care setting. In addition, Ranucci et al. [2], in a retrospective study of 9120 cardiac surgery patients revealed the increased preoperative Cr levels among the main determinants of late discharge from the ICU. Also, in the study of Atoui et al. [12] the history of renal failure was among the independent risk factors for extended cardiac surgery ICU hospitalization. Similar conclusions were reached by the studies of Bucerius et al. [13] and Ghotkar et al. [1].

However, in a recent retrospective study of 298 patients who underwent aortic arch surgery, the authors found that the increased serum Cr levels were associated with prolonged ICU stay, through the univariate analysis. Nevertheless, contrary to the finding of this study, the multivariate analysis did not indicate significant association between these two variables [5]. In addition, Wong et al. [14] correlated the high Cr levels postoperatively and not preoperatively with increased cardiac surgery ICU hospitalization.

We could find easily a logical interpretation regarding the previous correlation, based on the fact that patients with preexisting renal dysfunction or/and failure are characterised as high-risk patients with higher clinical severity and morbidity rates. In addition, these patients have higher incidence of postoperative complications, such as hemorrhage, reoperation, prolonged mechanical ventilation, strokes, and necessity for renal replacement therapy [15–17]. Moreover, the literature review reveals that the renal dysfunction during the preoperative period, among cardiac surgery patients, is a strong and independent indicator of increased in-hospital [18] and 30-day [15] mortality. Consequently, their stay for a longer period in the ICU in order to achieve clinical stability and to discharge from the critical care environment safely is rather reasonable.

Another important finding of this study was the significant correlation between the preoperative AF and the prolonged ICU-LOS. Similar to our result, Bucerius et al. [13], in a prospective study of 10759 patients undergoing CABG, observed that 20 variables (15 preoperative and 5 intraoperative), including the presence of AF preoperatively, have a strong association with prolonged ICU-LOS. Moreover, the authors of other studies [19, 20] found that the no-sinus rhythm or the rhythm disturbances, preoperatively, including AF, are significant predictors of prolonged stay in the cardiac surgery ICU.

The recent developments in invasive cardiology, the improvement of surgical techniques, and the improved pharmacological treatments have contributed to the application of surgical treatment among elder patients with advanced cardiovascular disease and significant comorbidity [13, 21]. Therefore, AF appears very frequently preoperatively in patients who are candidates for cardiac surgery, increasing the perioperative risk and the occurrence of postoperative complications [21]. Preoperative AF significantly worsens the heart functional status, increasing the incidence of postoperative complications, such as delirium, stroke, and low cardiac output syndrome, which lead to negative healthcare patient outcomes, including higher mortality and prolonged ICU and in-hospital stay [21–23]. In addition, a point of great interest is that while almost 30% of patients undergoing cardiac surgery will develop postoperative AF, if patients have history of AF (preoperative AF), this probability becomes almost 2 times greater (60%) [24, 25]. The above data in conjunction with the correlation between the postoperative AF and the prolonged cardiac surgery ICU-LOS [20] allow us to interpret the association of the preoperative AF with the prolonged ICU hospitalization, a finding which was revealed by our study.

In addition, via the multivariate analysis, we found that patients with high EuroSCORE have significantly greater probability to stay longer in the ICU than patients with lower perioperative risk. In line with our results, Giakoumidakis et al. [3], in a recent study of 313 consecutive cardiac surgery patients, concluded that there is a strong association between high perioperative risk and the prolonged ICU-LOS. In particular, patients with high logistic EuroSCORE values had an almost 1.9 times greater probability to stay in the ICU longer than 2 days. Several studies have reached to similar findings, revealing a positive association between increased perioperative risk (high EuroSCORE values) and the increased cardiac surgery ICU-LOS [10, 26, 27]. In addition, several investigators using risk stratification models other than EuroSCORE, such as Parsonnet Score, noted, in line with our findings, strong evidence between the high perioperative risk and the increased ICU-LOS [6, 12, 28].

The above correlation could be justified by the fact that variables, which are included in EuroSCORE, constitute significant risk factors and predictors of the increased hospitalization in the critical care environment. Older age, female gender, chronic pulmonary disease, extracardiac arteriopathy, neurological dysfunction, high preoperative Cr levels, previous cardiac surgery, unstable angina, poor left ventricular ejection fraction, recent myocardial infarction, emergency operation and procedures other than isolated coronary artery bypass grafting are parameters which are included in EuroSCORE model and simultaneously have been independently and separately correlated with increased ICU-LOS [3]. Indeed, a recent systematic review and validation study of the performance of 14 ICU-LOS prediction models among cardiac surgery patients revealed that the EuroSCORE model has significantly high performance in terms of discrimination, accuracy, and calibration. Among the 14 prediction models, only the Parsonnet score has greater performance than the EuroSCORE [6]. These data indicate that EuroSCORE is a valid and reliable tool for predicting the cardiac surgery ICU-LOS.

Moreover, we identified the significant correlation of one intraoperative variable with the prolonged ICU-LOS. In particular, patients with mean serum glucose levels >130 mg/dL, intraoperatively, had greater probability to stay longer in the ICU. In accordance with our results, Ouattara et al. [29] in a prospective cohort study of 200 diabetic patients undergoing coronary artery bypass grafting concluded that the poor glycemic control intraoperatively is correlated with significant increase in the ICU-LOS. Hyperglycemia during the perioperative period has been associated with negative patient outcome and constitutes an independent risk factor for higher morbidity and mortality [30]. Moreover, the poor glycemic control has been associated with postoperative complications, such as sternal wound infection, impaired wound healing, adverse renal and pulmonary events, increased incidence of AF and myocardial infarction [29, 31–35]. These data could interpret the prolonged ICU-LOS of these patients.

However, in contrary to the findings of the present study, the literature review shows that many investigators have not achieved to correlate the intraoperative hyperglycemia with the prolonged ICU-LOS among cardiac surgery patients [35–38]. In addition, Lazar et al. [32] in a controlled randomized trial of 141 diabetics patients who underwent coronary artery bypass graft surgery correlated the inadequate intra- and postoperative glycemic control with increased total postoperative in-hospital stay and not ICU stay separately. The significant shortage of randomized controlled trials and the different definitions of the perioperative hyperglycemia in conjunction with the different intensity and duration of the applied serum glucose therapy do not allow safe conclusions and could justify the inconsistence of the literature findings [39, 40].

Finally, the median ICU-LOS of our study was 2 days, a finding that is in line with the international literature, based on the fact that most researchers have demonstrated the 2 days as the cutoff point for the increased cardiac surgery ICU-LOS [3, 10].

## 5. Limitations

Our study allows us to reach significant conclusions regarding the preoperative and intraoperative factors that could influence the ICU-LOS among cardiac surgery patients. In addition, to the best of our knowledge, this study is among the few Greek studies aiming to investigate parameters that could predict the length of ICU stay and consequently to contribute to administrative decisions regarding the plan of the operative list, the provided care, and the ICU resources' allocation.

However, our study has some limitations. Firstly, the small sample size in conjunction with the fact that this study was carried out in one cardiac surgery centre limits the generalization of the finding to the general cardiac surgery population and affects the external validity of the study. Secondly, the ICU-LOS is often a subjective outcome that affected, except from clinical (e.g., patient severity, complications) and by nonclinical factors, such as the ICU and the ward capacity of the institution, the opinion of the intensive care specialists or a busy operation schedule [10]. Moreover some hospitals are more comfortable transferring patients out to a lower acuity nursing environment than other hospitals [3]. Finally, the different cut-off points of the prolonged ICU-LOS impede the ability to compare the findings of the corresponding international literature. Further research is needed to identify more predictive variables for the identification of patients at risk for prolonged cardiac surgery ICU hospitalization.

## 6. Conclusion

Demographic and clinical preoperative and intraoperative patient characteristics are significant predictors of the ICU-LOS among cardiac surgery patients. Patients with renal dysfunction, history of AF, high EuroSCORE values, and intraoperative hyperglycemia seem to have significantly greater probability of staying for more than 2 days in the intensive care environment. The early recognition of the nonmodified parameters (history of renal dysfunction and AF, high EuroSCORE) could lead to the early identification of patients at risk. In addition, the management of the modified predictor (intraoperative hyperglycemia), through the maintenance of normoglycemia during the operation, could erase a significant risk factor for prolonged ICU-LOS in the cardiac surgery setting.

Nursing administrators may use this knowledge for the appropriate use of the limited ICU, human and financial, resources. In addition, they have the opportunity for a more efficient and effective planning of the operative list, selecting low-risk patients in periods with significant restriction of the ICU beds availability. This chance has great importance for healthcare systems, such as the Greek, that are characterized by limited resources, nursing staff and ICU-beds shortage, mainly within the public sector.

## Figures and Tables

**Table 1:** Demographic and perioperative sample characteristics.

	*n* (%)	Median (IR)
Gender		
Male	102 (68.0)	
Female	48 (32.0)	
Age (years)		66 (15)
≤66	78 (52.0)	
>66	72 (48.0)	
BMI		27.6 (8.4)
Normal	49 (32.7)	
Overweight/obese	101 (67.3)	
Chronic pulmonary disease		
No	123 (82.0)	
Yes	27 (18.0)	
Diabetes		
No	101 (67.3)	
Yes	49 (32.7)	
Type of surgery		
Coronary artery bypass grafting (Type I)	67 (44.7)	
Valve disease repair (Type II)	41 (27.3)	
Aorta aneurysm repair	17 (11.3)	
Types I and II	18 (12.0)	
Other^∗^	7 (4.6)	
Preoperative serum Cr (mg/dL)		1 (0.4)
≤1.4	124 (82.7)	
>1.4	26 (17.3)	
Mean intraoperative blood glucose (mg/dL)		130 (36)
≤130	78 (52.0)	
>130	72 (48.0)	
Preoperative IABP		
No	146 (97.3)	
Yes	4 (2.7)	
CPB		
No	16 (10.7)	
Yes	134 (89.3)	
EFLV (%)		55 (15)
≤55	105 (70.0)	
>55	45 (30.0)	
Duration of surgery (min)		250 (110)
≤250	76 (50.7)	
>250	74 (49.3)	
Duration of CPB (min)		125 (69)
≤125	76 (50.7)	
>125	74 (49.3)	
Preoperative AF		
No	133 (88.7)	
Yes	17 (11.3)	
Emergency		
No	135 (90.0)	
Yes	15 (10.0)	
Ischemic time (min)		78 (54)
≤78	76 (50.7)	
>78	74 (49.3)	
RBC		
No	96 (64.0)	
Yes	54 (36.0)	
Logistic EuroSCORE (%)		5.4 (7.3)
≤5.4	75 (50.0)	
>5.4	75 (50.0)	
30-day mortality		
Alive	130 (86.7)	
Nonalive	20 (13.3)	
ICU-LOS (days)		2 (3)
≤2	76 (50.7)	
>2	74 (49.3)	

AF: atrial fibrillation, BMI: body mass index, CPB: cardiopulmonary bypass, Cr: creatinine, EFLV: ejection fraction of the left ventricle, EuroSCORE: European system for cardiac operative risk evaluation, IABP: intraaortic balloon pump, ICU: intensive care unit, LOS: length of stay, RBCs: red blood cells.

^∗^Another category refers to 7 patients who have undergone the closure of an atrial septal defect or a valve surgery in conjunction with aorta aneurysm repair.

**Table 2:** Bivariate analysis between the ICU-LOS and independent variables.

	ICU-LOS (days)		*P*-value
	≤2	>2
	*n* (%)	*n* (%)
Gender			0.48^∗^
Male	51 (50.0)	51 (50.0)	
Female	27 (56.3)	21 (43.8)	
Age (years)			0.89^∗^
≤66	41 (52.6)	37 (47.4)	
>66	37 (51.4)	35 (48.6)	
BMI			0.22^∗^
Normal	29 (59.2)	20 (40.8)	
Overweight/obese	49 (48.5)	52 (51.5)	
Chronic pulmonary disease			0.2^∗^
No	67 (54.5)	56 (45.5)	
Yes	11 (40.7)	16 (59.3)	
Diabetes			0.6^∗^
No	54 (53.5)	47 (46.5)	
Yes	24 (49.0)	25 (51.0)	
Type of surgery			0.06^∗^
Coronary artery bypass grafting (Type I)	40 (59.7)	27 (40.3)	
Valve disease repair (Type II)	23 (56.1)	18 (43.9)	
Aorta aneurysm repair	6 (35.3)	11 (64.7)	
Types I and II	5 (27.8)	13 (72.2)	
Other^∗∗∗^	4 (57.1)	3 (42.9)	
Preoperative serum Cr (mg/dL)			0.005*
≤1.4	71 (57.3)	53 (42.7)	
>1.4	7 (26.9)	19 (73.1)	
Mean intraoperative blood glucose (mg/dl)			0.002*
≤130	50 (64.1)	28 (35.9)	
>130	28 (38.9)	44 (61.1)	
Preoperative IABP			0.05**
No	78 (53.4)	68 (46.6)	
Yes	0 (0.0)	4 (100.0)	
CPB			0.72^∗^
No	9 (56.3)	7 (43.8)	
Yes	69 (51.5)	65 (48.5)	
EFLV			0.1^∗^
≤55	50 (47.6)	55 (52.4)	
>55	28 (62.2)	17 (37.8)	
Duration of surgery (min)			0.002*
≤250	49 (64.5)	27 (35.5)	
>250	29 (39.2)	45 (60.8)	
Duration of CPB (min)			**<**0.001*
≤125	51 (67.1)	25 (32.9)	
>125	27 (36.5)	47 (63.5)	
Preoperative AF			0.003*
No	75 (56.4)	58 (43.6)	
Yes	3 (17.6)	14 (82.4)	
Emergency			0.002*
No	76 (56.3)	59 (43.7)	
Yes	2 (13.3)	13 (86.7)	
Ischemic time (min)			0.073^∗^
≤78	45 (59.2)	31 (40.8)	
>78	33 (44.6)	41 (55.4)	
RBC			0.016*
No	57 (59.4)	39 (40.6)	
Yes	21 (38.9)	33 (61.1)	
Logistic EuroSCORE (%)			**<**0.001*
≤5.4	52 (69.3)	23 (30.7)	
>5.4	26 (34.7)	49 (65.3)	

AF: atrial fibrillation, BMI: body mass index, CPB: cardiopulmonary bypass, Cr: creatinine, EFLV: ejection fraction of the left ventricle, EuroSCORE: European system for cardiac operative risk Evaluation, IABP: intraaortic balloon pump, ICU: intensive care unit, LOS: length of stay, RBCs: red blood cells.

^∗^Chi-square test

^∗∗^Fisher's exact test

^∗∗∗^Another category refers to 7 patients who have undergone the closure of an atrial septal defect or a valve surgery in conjunction with aorta aneurysm repair.

**Table 3:** Logistic regression analysis (odds ratios and 95% confidence intervals) with ICU-LOS as dependent variable.

	ICU-LOS (days)			
	Crude OR (95% CI)	*P*-value	Adjusted^∗^ OR (95% CI)	*P*-value
Preoperative serum Cr (mg/dL)		0.007		**0.049**
≤1.4	1.0		1.0	
>1.4	3.6 (1.4–9.3)		3.0 (1.0–8.7)	
Mean intraoperative blood glucose (mg/dL)		0.002		**0.004**
≤130	1.0		1.0	
>130	2.8 (1.5–5.4)		3.0 (1.4–6.4)	
Preoperative AF		0.006		**0.012**
No	1.0		1.0	
Yes	6.0 (1.7–22.0)		6.3 (1.5–26.4)	
Logistic EuroSCORE (%)		<0.001		**0.017**
≤5.4	1.0		1.0	
>5.4	4.3 (2.2–8.4)		2.6 (1.2–5.6)	

AF: atrial fibrillation, CI: confidence interval, Cr: creatinine, EuroSCORE: European system for cardiac operative risk evaluation, ICU: intensive care unit, LOS: length of stay, OR: odds ratio.

^∗^Adjusted for gender, age, BMI, chronic pulmonary disease, diabetes, type of surgery, preoperative IABP, CBP, EFLV, duration of surgery, duration of cardiopulmonary bypass, emergency, ischemic time, and RBC.
